# Heterogeneity of subsets in glioblastoma mediated by Smad3 palmitoylation

**DOI:** 10.1038/s41389-021-00361-8

**Published:** 2021-10-27

**Authors:** Xiaoqing Fan, Junqi Fan, Haoran Yang, Chenggang Zhao, Wanxiang Niu, Zhiyou Fang, Xueran Chen

**Affiliations:** 1 Anhui Province Key Laboratory of Medical Physics and Technology, Institute of Health and Medical Technology, Hefei Institutes of Physical Science, Chinese Academy of Sciences, No. 350, Shushan Hu Road, Hefei, Anhui 230031, China. MOE Key Laboratory for Membraneless Organelles and Cellular Dynamics, University of Science & Technology of China, No. 96, Jin Zhai Road, Hefei, Anhui 230027 China; 2 University of Science and Technology of China, No. 96, Jin Zhai Road, Hefei, Anhui 230031 China; 3 Department of Anesthesiology, The First Affiliated Hospital of USTC, Division of Life Sciences and Medicine, University of Science and Technology of China (USTC), No. 17, Lu Jiang Road, Hefei, Anhui 230001 China; 4 Department of Laboratory Medicine, Hefei Cancer Hospital, Chinese Academy of Sciences, No. 350, Shushan Hu Road, Hefei, Anhui 230031 China

**Keywords:** Cancer stem cells, CNS cancer, Targeted therapies

## Abstract

Glioblastoma (GBM) is the most common and deadly of the primary intracranial tumors and is comprised of subsets that show plasticity and marked heterogeneity, contributing to the lack of success in genomic profiling to guide development of precision medicine for these tumors. In this study, a mutation in isocitrate dehydrogenase 1 was found to suppress the transforming growth factor-beta signaling pathway and E2F4 interacted with Smad3 to inhibit expression of mesenchymal markers. However, palmitoylation of Smad3 mediated by palmitoyltransferase ZDHHC19 promoted activation of the transforming growth factor-beta signaling pathway, and its interaction with EP300 promoted expression of mesenchymal markers in the mesenchymal subtype of GBM. Smad3 and hypoxia-inducible factor 1-alpha may be important molecular targets for treatment of glioma because they appear to coordinate the basic aspects of cancer stem cell biology.

## Introduction

Human glioblastoma (GBM) is the most common and deadly of the primary intracranial tumors and its treatment continues to be merely palliative [1, 2]. Treatment targeting a single molecular pathway is ineffective for GBM because of its marked intratumoral and intertumoral heterogeneity (including cellular and molecular complexity) [3, 4]. GBM contains glioblastoma stem cells (GSCs) that are highly resistant to radiotherapy and chemotherapy, which results in high recurrence rates [5, 6]. Targeting GSCs and identifying new markers are key issues in the development of innovative strategies to eradicate GBM.

Recent improvements in gene technology and application of whole-genome research have allowed identification of four molecular subtypes of GBM (namely, classical, proneural, neural, and mesenchymal), in patients based on The Cancer Genome Atlas (TCGA) [7, 8]. The neural subtype is now considered an artefact because this signature is thought to reflect the presence of normal neurons that reside within the tumor [7]. Classical GBM has a characteristic profile of highly proliferative cells and is responsive to conventional radiotherapy and chemotherapy, likely because the p53 DNA damage response is intact in these patients [7]. Isocitrate dehydrogenase 1 (IDH1) is the gene most commonly mutated in the proneural subtype of GBM [8, 9]. The mesenchymal subtype of GBM has a particularly prognosis that is characterized by an early tumor recurrence through therapeutic failure. Hyperactive transforming growth factor-beta (TGF-β) signaling could be a signature event in mesenchymal GBM, leading to dysregulation of downstream targets and contributing to malignant transformation [10, 11]. TGF-β increases glioma-initiating cell self-renewal via induction of leukemia inhibitor factor in human GBM and promotes proliferation via induction of platelet-derived growth factor-beta (PDGF-β) in gliomas with an unmethylated PDGF-β gene [12–14]. The plasticity of the transcription group in GBM allows transition from the proneural subgroup to the mesenchymal subgroup after cytotoxic treatment, and genomic analysis has not been successful in guiding the development of precision medicine for GBM [15, 16]. Therefore, there is an urgent need to identify therapeutic targets and novel biomarkers for these subsets and to investigate the regulatory mechanisms that are responsible for different GBM subsets considering GBM-specific niche contexts. In particular, activation of the TGF-β/Smad signaling pathway in the various subtypes of GBM and the role of this pathway in the transition of molecular subtypes are poorly understood.

Protein S-palmitoylation is a two-sided post-translational modification of proteins with fatty acids regulated by protein acyltransferases, which are characterized by a conserved Asp-His-His-Cys (DHHC) catalytic domain [17, 18]. Abnormalities in protein palmitoylation and dysregulation of DHHC proteins are involved in the development and progression of cancer [19–21]. ZDHHC5 palmitoylates EZH2 and modulates its methylation status in p53-mutant glioma [22], and inhibition of ZDHHC20 palmitoyltransferase results in dependence of cancer cells on the epidermal growth factor receptor for survival signals [23].

Here, we demonstrate that IDH1 mutation suppresses the TGF-β signaling pathway and that E2F4 interacts with Smad3 to inhibit expression of mesenchymal markers. However, Smad3 palmitoylation mediated by palmitoyltransferase ZDHHC19 promotes activation of the TGF-β signaling pathway, and its interaction with EP300 promotes expression of mesenchymal markers in the mesenchymal subtype of GBM.

## Results

### IDH1 knockdown elevates HIF-1α and decreases TGF-β1 in proneural GBM cells

**Fig. 1:**
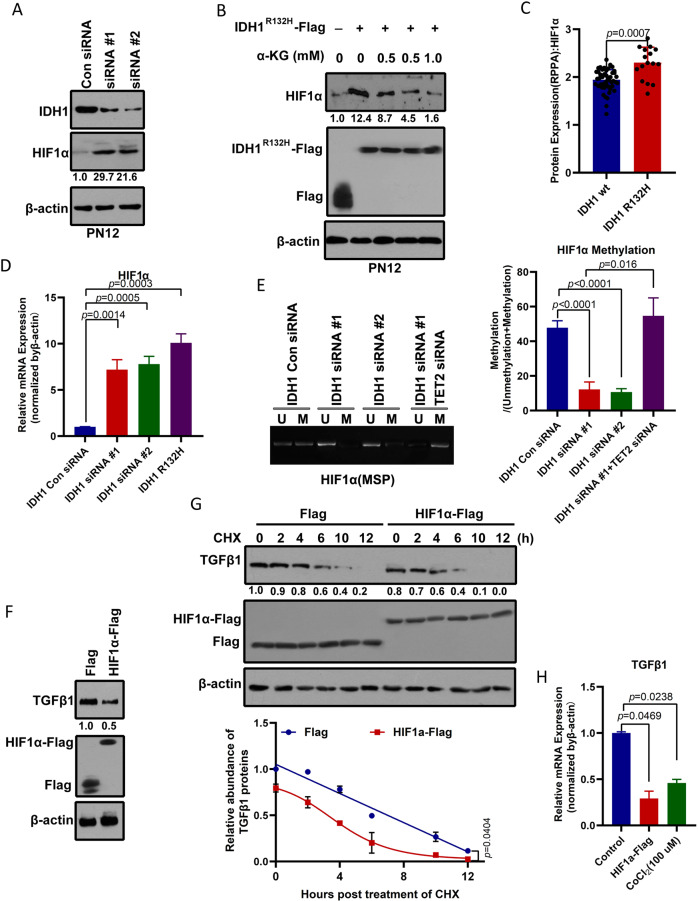
IDH1 mutation mediates induction of HIF-1α and TGF-β1 protein stability in proneural GSCs. **A** IDH1 knockdown elevates HIF-1α levels in the proneural GSC subtype, PN12. IDH1 and HIF-1α protein levels were determined by western blotting from PN12 GSCs transduced with control or siRNA targeting IDH1. The relative intensity (HIF-1α/β-actin) was quantified and indicated below. **B** A cell-permeable α-KG derivative blocks induction of HIF-1α in cells expressing IDH1 R132H. PN12 GSCs were transfected with IDH1 R132H, and different concentrations of octyl-α-KG ester were added to each transfected cell for 4 h. HIF-1α protein levels were assayed by western blotting. The relative intensity (HIF-1α/β-actin) was quantified and is indicated below. **C** Analysis of reverse-phase protein array expression data for proneural GBM from The Cancer Genome Atlas containing data for 64 tumor samples. HIF-1α protein levels were upregulated in IDH1 R132H mutant GBM (*n* = 16) when compared with wild-type IDH1 GBM (*n* = 48). Differences between groups were examined for statistical significance by the Student’s *t*-test using GraphPad Prism software, and the log-rank *p*-value was used to determine statistical significance, without multiple testing correction. **D** IDH1 knockdown or mutant IDH1 increases HIF-1α mRNA. After PN12 GSCs were transfected with control or siRNA targeting IDH1 or with the IDH1 R132H mutant, expression of HIF-1α mRNA was measured by quantitative polymerase chain reaction. **E** IDH1 knockdown reduces methylation of HIF-1α. Methylation-specific PCR (MSP) measurement of DNA methylation levels of the HIF-1α gene in PN12 GSCs. M and U represent amplification of methylated and unmethylated portions, respectively. Quantification of MSP band density for methylated alleles (closed bars) and unmethylated alleles (open bars) is shown. **F** Overexpression of HIF-1α reduced TGF-β1 levels in proneural GSCs, PN12. HIF-1α and TGF-β1 protein levels were determined by western blotting from PN12 GSCs transduced with control or Flag-tagged HIF-1α. The relative intensity (TGF-β1/β-actin) was quantified and is indicated below. **G** HIF-1α reduces the stability of TGF-β1 protein. PN12 GSCs were transfected with Flag-tagged HIF-1α for 24 h and then incubated with cyclohexidine 200 μg/ml, after which the cells were harvested at the indicated times for Western blot analysis. Relative TGF-β1 protein levels normalized to β-actin are presented relative to the level (set as 1.0) at 0 h post-CHX treatment (upper panel). **H** HIF-1α decreases expression of TGF-β1 mRNA. After PN12 was transfected with Flag-tagged HIF-1α or treated with CoCl_2_ (100 μmol/l) for 24 h, expression of TGF-β1 mRNA was measured by quantitative polymerase chain reaction. Analysis of variance coupled with Dunnett’s test (two-sided) was used for post hoc comparisons. *p < 0.05 versus the control group. GBM glioblastoma, GSCs glioblastoma stem cells, HIF-1α hypoxia-inducible factor 1-alpha, IDH1 isocitrate dehydrogenase 1.

Notably, IDH1 mutations were identified in proneural GBM. Tumor-derived IDH1 mutations impair formation of the enzyme product, α-ketoglutaric acid (α-KG). α-KG is required by prolyl hydroxylases, which are enzymes that hydroxylate and promote the degradation of hypoxia-inducible factor 1-alpha (HIF-1α) [9]. We found that HIF-1α protein levels in the proneural (PN12) subtype of GBM stem cells were elevated in response to IDH1 knockdown (Fig. 1A). Similarly, overexpression of the IDH1 R132H mutant increased HIF-1α protein levels in PN12 GSCs (Figs. 1B and S1A). However, octyl-α-KG suppressed induction of HIF-1α caused by overexpression of the IDH1 R132H mutant in PN12 GSCs (Fig. 1B and Fig. S1A). Analysis of reverse-phase protein array data from TCGA samples showed an increase in the level of HIF-1α in IDH1 R132H mutant GBM in comparison with that in wild-type IDH1 GBM (Fig. 1C). IDH1 knockdown or overexpression of the IDH1 R132H mutant-induced HIF-1α mRNA (Fig. 1D) might be due to aberrant methylation of HIF-1α (Fig. 1E). Moreover, TET2 knockdown would recover the decrease in methylation of HIF-1α mediated by IDH1 knockdown. Therefore, we concluded that a reduction in IDH1 activity produces a reduction in the α-KG level, which in turn leads to methylation and stabilization of HIF-1α.

Furthermore, the protein expression of TGF-β1 was significantly reduced in response to overexpression of HIF-1α in proneural (PN12) cells (Fig. 1F). Notably, knockdown or mutation of IDH1 did not affect the TGF-β1 mRNA level (Fig. S1B) and only led to a decrease in TGF-β1 protein (Fig. S1C). We also monitored the degradation kinetics of endogenous TGF-β1 protein following overexpression of HIF-1α using the cycloheximide chase assay. The TGF-β1 protein level decreased at the beginning of treatment with cycloheximide, and its half-life was markedly shorter in cells that overexpressed HIF-1α than in control cells (2.5 h vs 5.0 h; Fig. 1G). Indeed, the decrease in TGF-β1 transcription in response to hypoxia was further confirmed by stabilizing HIF-1α with CoCl_2_ (100 μmol/l) and overexpression of HIF-1α (Fig. 1H). Thus, we found that HIF-1α regulates the transcription activity and stability of TGF-β1 protein. Overall, IDH1 mutation promotes higher HIF-1α levels in proneural GBM, which in turn can lead to a reduction in TGF-β1.

### Smad3 constitutes an important effector in both proneural and mesenchymal GSCs

**Fig. 2:**
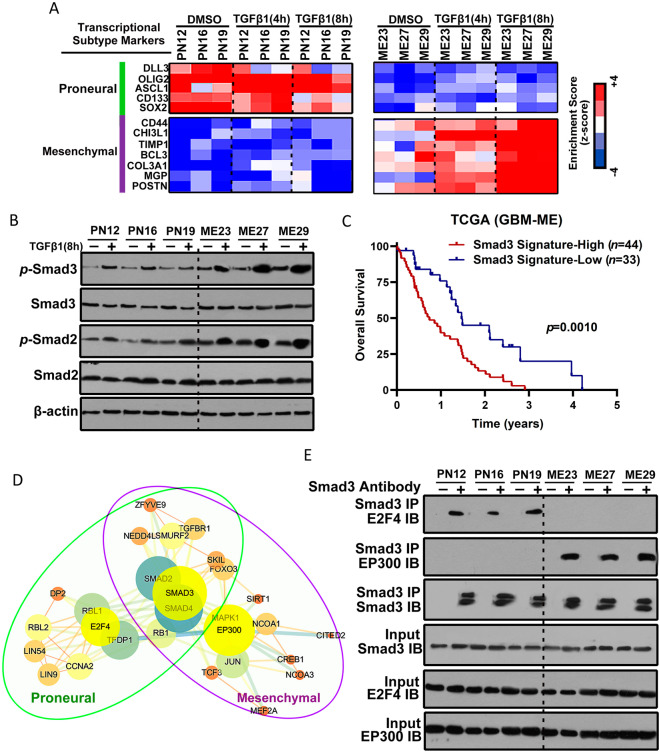
Smad3 constitutes an important effector in both proneural and mesenchymal GSCs. **A** Heatmap showing the molecular subtype marker expression in proneural GSCs (PN12, PN16, and PN19), and mesenchymal GSCs (ME23, ME27, and ME29) with or without TGF-β1 treatment for 4 or 8 h. Proneural markers: DLL3, OLIG2, ASCL1, CD133, and SOX2. Mesenchymal markers: CD44, CHI3L1, TIMP1, BCL3, COL3A1, MGP, and POSTN. Z-scores were calculated from the △Ct values obtained in the quantitative polymerase chain reaction analysis. **B** Western blotting analysis of SMAD2, *p*-SMAD2, SMAD3, and *p*-SMAD3 expression in proneural GSCs (PN12, PN16, and PN19) and mesenchymal GSCs (ME23, ME27, and ME29) with or without TGF-β1 treatment for 8 h. **C** Kaplan–Meier survival curves for patients with mesenchymal GBM and a high Smad3 gene signature (*n* = 44) and those with a low Smad3 gene signature (*n* = 33). **D** Protein–protein interaction networks indicated by STRING and Cytoscape software show overlapping peaks in both proneural and mesenchymal GSCs and also identify those that are unique to a particular subtype of GSCs or shared between the proneural and mesenchymal GSCs. **E** Lysates from proneural GSCs and mesenchymal GSCs were subjected to immunoprecipitation using anti-Smad3 antibody and then immunoblotted with anti-E2F4 and anti-EP300 antibodies. GSCs glioblastoma stem cells.

In view of the plasticity of the GBM transcriptional subgroups, these results prompted us to speculate that activation or inactivation of the TGF-β/Smad-signaling pathway might regulate the transformation of proneural GSCs into mesenchymal GSCs. Treatment with TGF-β1 did not change the levels of mesenchymal markers in proneural GSCs, although the expression levels of these markers were slightly reduced (Figs. 2A and S2A). However, the longer the duration of treatment with TGF-β1, the higher the gene expression profiles of mesenchymal markers in mesenchymal GSCs (Figs. 2A and S2A).

The functions of the TGF-β signaling pathway in the GBM subgroups were investigated at the molecular level by western blotting, which allowed analysis of the phosphorylation status of Smad2 and Smad3 in proneural and mesenchymal GBM cells. We found that mesenchymal cell lines (ME23, ME27, and ME29) showed a higher degree of activation of the TGF-β pathway than proneural cell lines (PN12, PN16, and PN19). In particular, treatment with TGF-β1 activated the TGF-β pathway more strongly in mesenchymal cell lines than in proneural cell lines (Fig. 2B). Overall, we demonstrated that neither treatment with TGF-β1 nor activation of the TGF-β pathway could induce proneural to mesenchymal transition.

We then performed single-sample gene set enrichment analysis and found that survival was significantly shorter in patients with mesenchymal GBM and a higher Smad3 gene signature (a gene transcriptionally related to Smad3) than in those with mesenchymal GBM and a lower Smad3 signature (*p* = 0.001; Fig. 2C).

Although there was a difference in activation of Smad3 between the proneural and mesenchymal GSCs, this protein was partly co-expressed in both cell types, indicating a likely association. Next, we investigated the complexes of Smad3 with other proteins using the anti-Smad3 antibody in both proneural and mesenchymal GSCs, and the components of the complexes were identified using liquid chromatography/tandem mass spectrometry. We analyzed the overlapping peaks in both the proneural and mesenchymal GSCs and identified those that were unique to a particular subtype of GSCs and those that were common to both proneural and mesenchymal GSCs. The protein–protein interaction networks were identified using STRING and Cytoscape software. Notably, proneural GSCs contained a Smad3-E2F4 complex whereas mesenchymal GSCs contained a Smad3-EP300 complex (Fig. 2D). Consistent with these findings, immunoprecipitation analysis confirmed interaction between Smad3 and E2F4 in proneural GSCs and the presence of Smad3 and EP300 complexes in mesenchymal GSCs (Fig. 2E). Moreover, co-immunoprecipitation and pull down of Smad3 demonstrated competitive interaction of E2F4 and EP300 with Smad3 (Fig. S2B and S2C).

E2F4 is a transcription suppression factor [24] and histone acetyltransferase EP300 is a transcription cofactor [25]. Therefore, we hypothesized that E2F4 interacts with Smad3 to suppress the expression of mesenchymal markers in proneural GSCs and that EP300 competitively interacts with Smad3 to activate expression of mesenchymal markers in mesenchymal GSCs.

### Palmitoylation of Smad3 promotes its association with the plasma membrane and activation of the TGF-β pathway

**Fig. 3:**
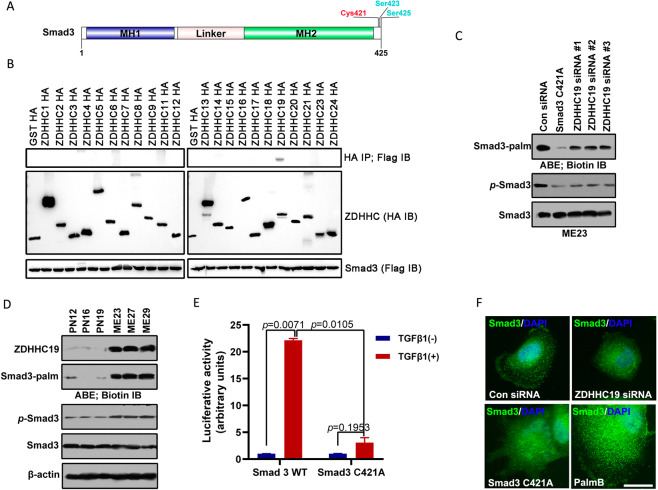
Palmitoylation of Smad3 mediated by ZDHHC19 promotes its association with the plasma membrane and activation of the TGF-β pathway. **A** Diagram showing the sites of palmitoylation of Smad3. CSS-Palm 4.0 software indicated that Cys421 was a potential palmitoylation site for Smad3. **B** HEK293 cells were co-transfected with constructs encoding Flag-Smad3 and HA-ZDHHCs in a 6-well plate. Cell lysates were harvested for immunoprecipitation by anti-Flag antibody and then for acyl–biotin exchange (ABE) and western blot analysis. The results of immunoblotting for total Smad3 and HA-ZDHHC proteins are shown. **C** Palmitoylation and phosphorylation of Smad3 in ME23 cells transfected with Smad3 C421A mutant constructs or infected with ZDHHC19 siRNA for 48 h. **D** Expression levels of ZDHHC19 and Smad3 and palmitoylation level of Smad3 in proneural and mesenchymal GSCs. **E** The Smad3 palmitoylation mutant suppressed activation of the TGF-β/Smad-signaling pathway. ME23 cells were stably transfected with (SBE)_4_-luc, which can be activated by TGF-β1. These ME23 cells were then transfected with Smad3 or Smad3 C421A and stimulated with 5 ng/ml TGF-β1 for 18 h starting 24 h after transfection. **F** Expression of Smad3 in ME23 GSCs (monolayer culture) transfected with ZDHHC19 siRNA or Smad3 C421A mutant plasmid or treated with the de-palmitoylation inhibitor, PalmB (1 μM) for 48 h was analyzed by immunofluorescence staining. Scale bar, 100 μm. GSCs glioblastoma stem cells.

Several recent studies have indicated that the DHHC (Asp-His-His-Cys)-S-acyltransferase protein family and its substrates play an important role in tumorigenesis, especially in the development and progression of glioma [22, 26, 27]. The CSS-Palm 4.0 software used in this study indicated that Smad3 has one potential palmitoylation site (Fig. 3A). To identify which protein S-acyltransferases were responsible for modification of Smad3, 23 HA-tagged ZDHHC protein S-acyltransferases were co-expressed with Flag-Smad3 (Fig. S3A). Smad3 palmitoylation was only detected following expression of ZDHHC4, 6, 11, 14, 19, and 23, with ZDHHC19 being the most efficient (Fig. 3B). Palmitoylation of Smad3 was substantially reduced in cells expressing C421A mutant Smad3 and diminished by silencing of ZDHHC19 (Figs. 3C and S3B). Notably, there was a positive association between activation of Smad3 and palmitoylation of Smad3 (Figs. 3C and S3B). The palmitoylation level of Smad3 was decreased by 2-BP (a palmitoylation inhibitor) and increased by PalmB (a de-palmitoylation inhibitor) (Fig. S3C). Furthermore, a Click-iT Labeling assay confirmed that Smad3 could be palmitoylated by ZDHHC19 (Fig. S3D). Consistent with these findings, ZDHHC19 expression levels and Smad3 palmitoylation levels were higher in mesenchymal cell lines than in proneural cell lines (Fig. 3D). Moreover, the Smad3 palmitoylation mutant was unable to activate the TGF-β/Smad-signaling pathway (Fig. 3E). Thus, ZDHHC19 expression levels and Smad3 palmitoylation levels were identified as markers for the mesenchymal or proneural subtype. We then assessed whether palmitoylation of Smad3 influenced its distribution/localization in GSCs. Both immunostaining and western blotting showed that GSCs transfected with the Smad3 C421A mutant or ZDHHC19 siRNA had reduced expression of Smad3 on the membrane surface and that those treated with the de-palmitoylation inhibitor PalmB maintained the same level of Smad3 in comparison with normal conditions (Figs. 3F and S3E). These findings indicate that palmitoylation of Smad3 helps Smad3 traffic in the membrane fractions and activates the TGF-β/Smad3 pathway.

### Inhibition of HIF-1α is effective in the proneural subtype and inhibition of Smad3 is effective in the mesenchymal subtype

**Fig. 4:**
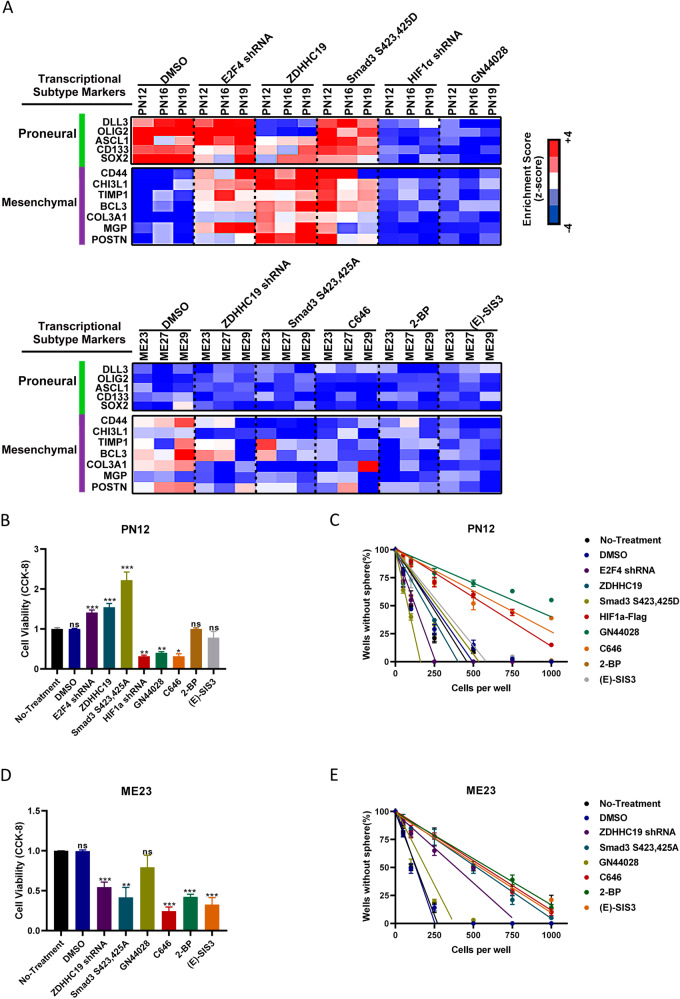
Inhibition of HIF-1α or Smad3 could reduce cell viability and self-renewal in the proneural and mesenchymal subtypes of GSCs. **A** Heatmap showing molecular subtype marker expression in proneural GSCs (PN12, PN16, and PN19), mesenchymal GSCs (ME23, ME27, and ME29), and proneural (or mesenchymal) GSCs infected with the indicated plasmids or shRNA or treated with the indicated inhibitors. HIF-1α inhibitor, GN44028, 12.5 μM. Palmitoylation inhibitor, 2-BP, 100 μM. Smad3 inhibitor, (E)-SIS3, 20 μM. EP300 inhibitor, C646, 15 μM. Z-scores were calculated from the △Ct values obtained in the quantitative polymerase chain reaction analysis. **B** Viability of proneural PN12 GSCs transfected with the indicated plasmids or shRNA or treated with GN44028, C646, 2-BP, or (E)-SIS3 for 48 h. **C** A serial dilution assay was used to determine the self-renewal capacity of PN12 GSCs transfected with the indicated plasmids or shRNA or treated with GN44028, C646, 2-BP, or (E)-SIS3. **D** Viability of mesenchymal ME23 GSCs transfected with the indicated plasmids or shRNA or treated with GN44028, C646, 2-BP, or (E)-SIS3 for 48 h. **E** A serial dilution assay was used to determine the self-renewal capacity of ME23 GSCs transfected with the indicated plasmids or shRNA or treated with GN44028, C646, 2-BP, or (E)-SIS3. GSCs glioblastoma stem cells, HIF-1α hypoxia-inducible factor 1-alpha.

Depletion of E2F4, upregulation of ZDHHC19, or activation of Smad3 reduced expression levels of proneural markers in proneural GSCs and increased those of mesenchymal markers to varying degrees (Fig. 4A). Moreover, both proneural and mesenchymal markers were reduced after depletion of HIF-1α or treatment with the HIF-1α inhibitor, GN44028 (Figs. 4A and S4). In mesenchymal GSCs, depletion of ZDHHC19 or inactivation of Smad3 reduced the expression levels of mesenchymal markers with no change in levels of proneural markers (Figs. 4A and S4). The mesenchymal GSCs displayed preferential sensitivity to treatment with C646 (an EP300 inhibitor), 2-BP (a palmitoylation inhibitor), or (E)-SIS3 (a Smad3 inhibitor), similar to the effect of ZDHHC19 depletion (Figs. 4A and S4).

To leverage our findings for clinical application, we examined the sensitivity of proneural GSC subtypes to HIF-1α inhibitors and the sensitivity of mesenchymal subtypes to palmitoylation inhibitors or Smad3 inhibitors. Notably, depletion of E2F4, upregulation of ZDHHC19, or activation of Smad3 increased the viability (Fig. 4B) and self-renewal (Fig. 4C) of proneural GSCs, whereas inactivation of HIF-1α blocked these effects. In mesenchymal GSCs, depletion of ZDHHC19, inactivation of Smad3, inactivation of EP300, or inhibition of palmitoylation could reduce cell viability (Fig. 4D) and self-renewal (Fig. 4E).

**Fig. 5:**
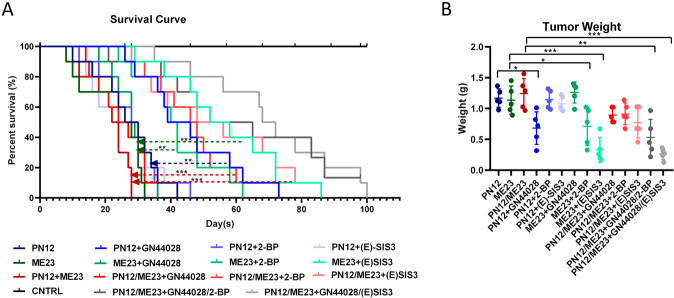
In vivo therapeutic efficacy of pharmacologic inhibitors of HIF-1α and Smad3 used alone or in combination in a single subtype model and a mixed glioblastoma model. **A** Kaplan–Meier survival curves for mice bearing a single subtype of tumor and mice bearing a mixed orthotopic tumor with PN12 and ME23 cells that were treated with 10 mg/kg GN44028, 7.5 mg/kg 2-BP, and 15 mg/kg (E)-SIS3 alone or in combination (*n* = 15 per group). Log-rank *p*-values were used to determine statistical significance. *p < 0.05. **B** Tumor weight in mice with a single subtype of tumor and mice with a mixed orthotopic tumor with PN12 and ME23 cells treated with 10 mg/kg GN44028, 7.5 mg/kg 2-BP, and 15 mg/kg (E)-SIS3 alone or in combination (*n* = 5 per group) after 6 weeks.

Next, we examined the therapeutic effects of HIF-1α, EP300, Smad3, and a palmitoylation inhibitor in mice bearing intracranial tumors derived from proneural or mesenchymal GSCs (Fig. 5). Inhibition of HIF-1α activity extended the survival rate of animals with implanted proneural GSCs more efficiently than in animals that were not treated with GN44028. Moreover, inhibition of palmitoylation or (E)-SIS3 injection resulted in inhibition of tumor growth and extended survival in all animals with implanted mesenchymal GSCs. We also investigated the effects of treatment with these inhibitors as monotherapy or in combination in xenografts containing a mixture of both proneural and mesenchymal GSCs in vivo. The results indicated that combinatorial inhibition of HIF-1α and Smad3 activity is an effective treatment strategy for heterogeneous subtypes of GBM. Moreover, both proneural and mesenchymal markers were reduced after treatment with the HIF-1α inhibitor, GN44028 (Fig. S5A). 2-BP or (E)-SIS3 could reduce the expression levels of mesenchymal markers with only a slight change in levels of proneural markers (Fig. S5A). These findings may be related to Smad3 palmitoylation or the phosphorylation level (Fig. S5B).

**Fig. 6:**
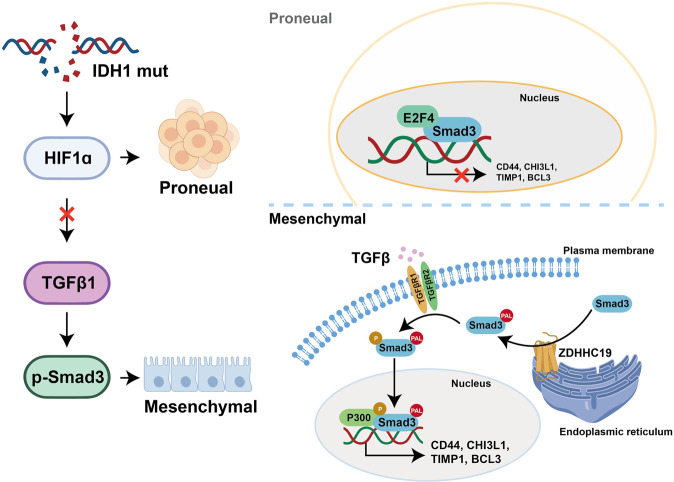
Schematic diagram. IDH1 mutation suppressed the TGF-β signaling pathway and E2F4 interacted with Smad3 to inhibit expression of mesenchymal markers in the proneural subtype of GBM. However, palmitoylation of Smad3 mediated by palmitoyltransferase ZDHCH19 promoted activation of the TGF-β signaling pathway and its interaction with EP300 to promote expression of mesenchymal markers in the mesenchymal subtype of GBM. GBM glioblastoma, IDH1 isocitrate dehydrogenase 1.

In summary, we found that in proneural GSCs, IDH1 mutation promotes higher HIF-1α levels and reduces expression of TGF-β1 and activation of the TGF-β signaling pathway. The transcription suppression factor E2F4 interacts with Smad3 to suppress the expression of mesenchymal markers. However, palmitoylation of Smad3 mediated by the palmitoyl transferase ZDHCH19 promotes its association with the cytoplasmic membrane and its S423,425 phosphorylation in mesenchymal GSCs. Finally, palmitoylated and phosphorylated Smad3 translocates into the nucleus and interacts with EP300 to promote expression of mesenchymal markers. Furthermore, combinatorial inhibition of HIF-1α and Smad3 activity is an effective treatment strategy for heterogeneous subtypes of GBM (Fig. 6).

## Discussion

Tumor cell heterogeneity is caused by variations in genetics, epigenetic cell states, and microenvironments. In this study, we found that IDH1 mutation-induced activation of HIF-1α and reduced expression of TGF-β1 in proneural GSCs and that Smad3 co-interacted with E2F4 to inhibit expression of mesenchymal markers. In mesenchymal GSCs, we identified a mechanism that could regulate activation of Smad3 through palmitoylation of the Smad3 C-terminal tail. Palmitoylation facilitates activation of Smad3 via membrane positioning and phosphorylation via TGF-β1/2. Blocking the activity of HIF-1α and that of Smad3 could be effective against the proneural and mesenchymal subtypes of GBM, respectively.

According to the molecular signature, GBM is divided into four subtypes (proneural, neural, classical, and mesenchymal), each of which has its own hereditary background. In one study, 41% of primary GBM cases were identified as the mesenchymal subtype and only 15% as the proneural subtype; however, the ratio of subtypes was the opposite for secondary GBM (mesenchymal, 15%; proneural, 44%) [28]. Mutations in IDH1 were found to be more common in the proneural subtypes. IDH1 gene mutations in gliomas exhibit two unique features: lack of loss of heterozygosity and lack of apparent inactivating mutations such as truncations or frame shifts [29]. Tumor-derived mutant IDH1 suppresses wild-type IDH1 mainly by forming a catalytically inactive heterodimer, leading to a decline in cellular α-KG [9]. This reduction is produced by a decrease in the activity of IDH1 at the α-KG level, which in turn can lead to stabilization of HIF-1α. Proneural GBMs are markers of mature vessels. IDH1-mutated gliomas also exhibit an increase in levels of vascular endothelial growth factor when compared with gliomas without IDH1 mutations of similar type and grade [30].

Moreover, we found that HIF-1α interferes with signal transduction of TGF-β1 by reducing mRNA and protein expression of TGF-β1 and by reducing Smad3 phosphorylation. However, these findings are not consistent with other reports. First, the TGF-β1/Smad3 signaling pathway has been found in hypoxia-activated fibroblasts and the chromatin immunoprecipitation assay identified the presence of hypoxic response elements in the TGF-β1 promoter in mesenchymal stem cells [31]. Second, a study in rodent mandibular osteoblasts found that expression of TGF-β1 was shown to have undergone a state of hypoxia, which gradually decreased when the duration of hypoxia was prolonged [32]. Therefore, it is obvious that HIF-1α can induce expression of TGF-β1 in several types of cells and at different stages of cell growth. However, HIF-1α can also inhibit the expression of TGF-β1. In human fetal osteoblasts, hypoxia could lead to decreased expression of TGF-β1 [33], which is consistent with our present findings. Reverse expression of the inhibitory signal transduction from HIF-1α to TGF-β1 expression and phosphorylation of Smad3 showed the self-correction between TGF-β1 and HIF-1α in GSCs.

Mesenchymal GBM also expresses markers for hypoxia in the microvasculature [16]. Activation of the TGF-β pathway was found to be strongly associated with the mesenchymal subtype [34, 35]. Therefore, in the mesenchymal subtype of GBM, there is another way to regulate the TGF-β signaling pathway. Palmitoylation is required for intracellular transport, cell membrane positioning, and phosphorylation of Smad3. The lower activation of the TGF-β signaling pathway may be accounted for by the defect in membrane localization of Smad3, which is observed in ZDHHC19-silenced proneural cells. Our findings suggest that intracellular transport and cell membrane positioning provide a platform for Smad3 to be phosphorylated by TGF-β1/2 at the plasma membrane and signal within endosomal compartments.

For proneural GSCs, pharmacologic blockade of HIF-1α activity by GN44028 and of Smad3 activity by the Smad3 inhibitor, (E)-SIS3 or a palmitoylation inhibitor, 2-BP, effectively suppressed tumor growth and extended survival rates in animals with xenografts. Combinatorial therapy achieved the most effective control of both mesenchymal and proneural tumors, although monotherapy alone targeted to Smad3 or HIF-1α hindered tumor growth near the detection limits. GBM cells can undergo transitions in molecular subtype in response to chemotherapy or radiotherapy and according to the microenvironment of the tumor, which may lead to differences in effects between in vitro and in vivo experiments using HIF-1α and Smad3 inhibitors.

In summary, we found that mutation of IDH1 and activation of HIF-1α are significant signatures in proneural cells whereas activation of the TGF-β/Smad3 signaling pathway is characteristic of the mesenchymal subtype. Thus, the activity of Smad3 and HIF-1α could represent the main molecular targets for treatment of gliomas because these proteins may coordinate the basic aspects of cancer stem cell biology. Further studies are needed to determine how the interconversion of different pools of GSCs may support adaptive resistance to targeted therapies.

## Materials and methods

### Reagents, antibodies, and plasmids

The α-KG (75890) and palmitate analog inhibitor 2-bromopalmitate (2-BP, 238422) were purchased from Sigma-Aldrich (St Louis, MO, USA). The HIF-1α inhibitor GN44028 (HY-110266), Smad3 inhibitor, (E)-SIS3 (HY-13013), and EP300 inhibitor C646 (HY-13823) were obtained from MedChemExpress (Monmouth Junction, NJ, USA).

ZDHHC19 (orb186631) was sourced from Biorbyt Ltd (Cambridge, UK). Smad3 (9523), E2F4 (40291), EP300 (54062), and β-actin (3700) were purchased from Cell Signaling Technology (Beverly, MA, USA). Flag-tag (F1804) and HA-tag (H9658) were obtained from Sigma-Aldrich.

Full-length *IDH1*, *HIF-1*α, or *Smad3* cDNA was cloned into a pcDNA3.0 expression vector (Invitrogen, Waltham, MA, USA) for the overexpression studies. IDH1 R132H, Smad3 C421A, Smad3 S423,425A, and Smad3 S423,425D mutant constructs were generated using the QuikChange Site-directed Mutagenesis Kit (Stratagene, San Diego, USA). All constructs were verified by DNA sequencing. Subsequently, the vector constructs were transfected into the cells using Lipofectamine™ 2000 transfection reagent according to the manufacturer’s instructions.

Cells were also transduced with pGFP-C-shLenti virus specks specifically for control shRNA or E2F4 shRNA (#TL313319; Origene, Rockville, MD, USA), HIF-1α shRNA (#TL320380, Origene), ZDHHC19 shRNA (#TL317857, Origene), or with pLenti-C-Myc-DDK-P2A-Purovirus species specifically for ZDHHC19 (#RC221741L3V, Origene). The cells were treated with puromycin (0.5 μg/ml; #A1113802, Thermo Fisher Scientific, Waltham, MA, USA) over 2 weeks to establish stable cell lines.

### Cell culture

Proneural GSCs (PN12, PN16, and PN19) and mesenchymal GSCs (MES23, MES27, and MES29) were isolated from surgical specimens and confirmed in an earlier study [26]. GSCs were cultured in neurobasal medium with B27 (Invitrogen), basic fibroblast growth factor (20 ng/ml), and epidermal growth factor (20 ng/ml). All the cell lines used in this study were subjected to short tandem repeat analysis and tested for mycoplasma, most recently in February 2021.

### Quantitative real-time polymerase chain reaction

Total RNA was isolated using a MiniBEST Universal RNA Extraction Kit (9767, TaKaRa, Kusatsu, Japan), and real-time polymerase chain reaction (PCR) was performed on a 7300 Cycler (Applied Biosystems, Waltham, MA, USA) using a Titanium One-Step real-time PCR kit (639504, Clontech Laboratories, Mountain View, CA, USA). Each sample was prepared in triplicate, and the levels of target gene expression were calculated using the 2^−△△Ct^ method, with β-actin serving as the internal control. The sequences of gene-specific primers used in the study were as follows: *DLL3*, 5′-GAG ACA CCC AGG TCC TTT GA-3′ and 5′-CAG TGG CAG ATG TAG GCA GA-3′; *OLIG2*, 5′-GAC AAG CTA GGA GGC AGT GG-3′ and 5′-CGG CTC TGT CAT TTG CTT CT-3′; *ASCL1*, 5′-CGG CCA ACA AGA TGA GT-3′ and 5′-TGG AGT TGG GGG AGA TG-3′; *CD133*, 5′-TTG TGG CAA ATC ACC AGG TA-3′ and 5′-TCG ATC TGT GAA CGC CTT G-3′; *SOX2*, 5′-CAC AAC TCG GAG ATC GCA A-3′ and 5′-CGG GGC CGG TAT TTA TAA TC-3′; *CD44*, 5′-AAG GTG GAG CAA ACA CAA CC-3′ and 5′-AGC TTT TTC TGC CCA CA-3′; *CHI3L1*, 5′-GAT AGC CTC CAA CAC CCA GA-3′ and 5′-AAT TCG GCC TTC ATT TCC TT-3′; *TIMP1*, 5′-TGA CAT CCG GTT CGT CTA CA-3′ and 5′-TGA TGT GCA AGA GTC CAT CC-3′; *HIF1α* forward 5′-CCT ATG TAG TTG TGG AAG TT TAT GC-3′ and reverse 5′-ACT AGG CAA TTT TGC TAA GAA TG-3′; *BCL3* forward 5′-CCC TAT ACC CCA TGA TGT GC-3′ and reverse 5′-GGT GTC TGC CGT AGG TTG GTT-3′; *COL3A* forward 5′-CCA GGG AGC TAA CGG TAT CAG-3′ and reverse 5′-CAGGGTTTCCATCTCTTCCA-3′; *MGP* forward 5′-CAC GAG CTC AAT AGG GAA GC-3′ and reverse 5′-CGC TTC CTG AAG TAG CGA TT-3′; *POSTN* forward 5′-GTC TTT GAG ACG CTG GAA GG-3′ and reverse 5′-AGA TCC GTG AAG GTG GTT TG-3′; and *β-actin*, 5′-AGA AAA TCT GGC ACC ACA CC-3′ and 5′-GGG GTG TTG AAG GTC TCA AA-3′.

### Immunoprecipitation, the ABE labeling method, and western blot analysis

Protein–protein interactions were detected using a Pierce Crosslink magnetic IP and Co-IP kit (Thermo Scientific). The immunoprecipitated and co-immunoprecipitated proteins were analyzed by sodium dodecyl sulfate-polyacrylamide gel electrophoresis (SDS-PAGE) and western blotting, respectively.

To determine the level of protein palmitoylation, an acyl-biotin exchange (ABE) assay was performed, as previously described [36]. Briefly, the immunoprecipitated beads were incubated for 1 h at 4 °C with wash buffer (50 mM Tris, pH 7.4, including 5 mM EDTA, 150 mM NaCl, and 1% Triton X-100) supplemented with 50 mM *N*-ethylmaleimide. Next, the beads were incubated with 1 M hydroxylamine (pH 7.4) for 1 h at room temperature and exposed to 0.5 μM 1-biotinamido-4-(4-[maleimidoethylcyclohexane]-carboxamido) butane (pH 6.2) for 1 h at 4 °C. Samples were analyzed by SDS-PAGE and immunoblotting.

Cell lysates were quantified by bicinchoninic acid assay, resolved by SDS-PAGE, and transferred onto a nitrocellulose blotting membrane (Bio-Rad Laboratories, Hercules, CA, USA) that was subsequently incubated with primary antibodies. Finally, the membranes were exposed to the corresponding horseradish peroxidase-conjugated secondary antibodies (Bio-Rad), and the bands were detected using enhanced chemiluminescence (Bio-Rad).

### Cell viability assays

Cell viability was determined using the CellTiter-Glo Luminescent Cell Viability Assay kit (Promega, Madison, WI, USA) according to the manufacturer’s instructions.

### In vitro tumorsphere formation

For suspension culture/tumorsphere formation, 50, 100, 250, 500, 750, and 1000 cells were seeded in 96-well plates containing 200 μl of complete neurobasal medium. After 10 days, the tumorspheres were measured and analyzed.

### Animal experiments

This component of the study was approved by the institutional review board of Hefei Cancer Hospital, Chinese Academy of Sciences (approval number, SWYX-Y-2020-08). All animal experiments were performed according to the guidelines of the Animal Use and Care Committees at the Hefei Institutes of Physical Science, Chinese Academy of Sciences (approval number, SWYX-DW-2020-02).

For the implantation study, a stereotactic method was utilized to inject a suspension of 500 PN12 or/and 500 ME23 GSCs in phosphate-buffered saline with high glucose into the hemi-striatum of 6-week-old BALB/c mice (female, 20–30 g) after anesthesia with ketamine (132 mg/kg) and xylazine (8.8 mg/kg). The following coordinates were used: dorsoventral, −3.5 mm; mediolateral, +2.5 mm; and anteroposterior, *r* = 0. After 1 week, an HIF-1α inhibitor (GN44028, 10 mg/kg), palmitoylation inhibitor (2-BP, 7.5 mg/kg), and/or Smad3 inhibitor [(E)-SIS3, 15 mg/kg] was administered orally by gavage. Finally, the tumor-bearing mice were sacrificed by injection of sodium pentobarbital (50 mg/kg) and analyzed.

### Patient dataset analysis and gene set enrichment analysis

The enrichment score was developed by single-sample gene set enrichment analysis (GSEA; ssGSEA: http://software.broadinstitute.org/cancer/software/genepattern#). The Smad3 signature was derived as previously described [37]. GSEA (https://www.broadinstitute.org/gsea/), a knowledge-based approach for interpreting genome-wide expression profiles, was performed as described elsewhere [38].

### Statistical analysis

All data are presented as the mean ± standard error of the mean. Differences between groups were examined for statistical significance by one-way analysis or the Student’s *t*-test using GraphPad Prism software (GraphPad Software Inc., La Jolla, CA, USA). Correlation coefficients were calculated using Excel software. Kaplan–Meier survival curves were generated using GraphPad Prism software, and the log-rank *p*-value was used to determine statistical significance. All experiments were repeated in each specimen presented in at least three biological duplicates (patient-derived xenograft cell models) with technical triplicates.

## Supplementary information

Supplementary Figures
