# Correction: Heterogeneity of subsets in glioblastoma mediated by Smad3 palmitoylation

**DOI:** 10.1038/s41389-021-00368-1

**Published:** 2021-11-12

**Authors:** Xiaoqing Fan, Junqi Fan, Haoran Yang, Chenggang Zhao, Wanxiang Niu, Zhiyou Fang, Xueran Chen

**Affiliations:** 1 Anhui Province Key Laboratory of Medical Physics and Technology, Institute of Health and Medical Technology, Hefei Institutes of Physical Science, Chinese Academy of Sciences, No. 350, Shushan Hu Road, Hefei, Anhui 230031 China; 2 University of Science and Technology of China, No. 96, Jin Zhai Road, Hefei, Anhui 230031 China; 3 Department of Anesthesiology, The First Affiliated Hospital of USTC, Division of Life Sciences and Medicine, University of Science and Technology of China (USTC), No. 17, Lu Jiang Road, Hefei, Anhui 230001 China; 4 Department of Laboratory Medicine, Hefei Cancer Hospital, Chinese Academy of Sciences, No. 350, Shushan Hu Road, Hefei, Anhui 230031 China

**Keywords:** Cancer stem cells, CNS cancer, Targeted therapies

Correction to: *Oncogenesis* 10.1038/s41389-021-00361-8, published online 27 October 2021

Due to a processing error the affilations to all authors were given incorrectly in the pdf file.

The original article has been corrected.

