# Supplementary material for: Heterogeneity of subsets in glioblastoma mediated by Smad3 palmitoylation
Source: Oncogenesis. 2021 Oct 27;10(10):72. doi: 10.1038/s41389-021-00361-8 (PMC8551152; doi:10.1038/s41389-021-00361-8)
Supplement: Supplementary file 1 — Supplementary Figures [file 41389_2021_361_MOESM1_ESM.pdf]

Supplementary Figures

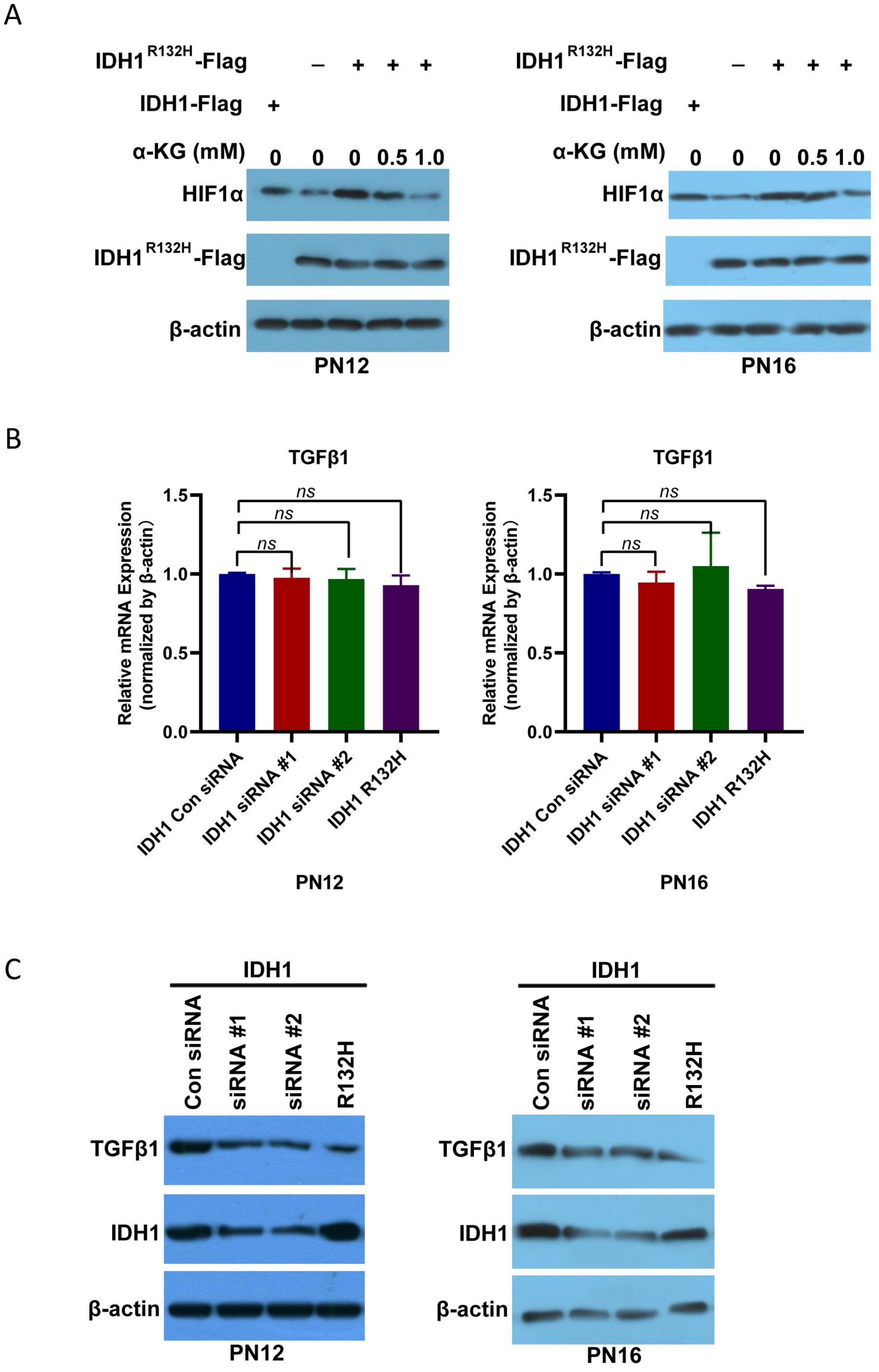

Figure S1. IDH1 regulated expression levels of HIF-1α and TGF-β1. (**A**) Alpha-ketoglutaric acid blocks induction of HIF-1α in cells expressing IDH1 R132H. PN12 (left) or PN16 (right) GSCs were transfected with wild-type IDH1 or IDH1 R132H, and different concentrations of octyl-α-KG ester were added to each type of transfected cell for 4 h. HIF-1α protein levels were assayed by Western blotting. (**B**) IDH1 knockdown or mutant does not affect TGF-β1 mRNA. After PN12 (left) or PN16 (right) GSCs were transfected with control or siRNA targeting IDH1 or with IDH1 R132H mutant, expression of TGF-β1 mRNA was measured by quantitative polymerase chain reaction. (**C**) IDH1 knockdown or mutant reduces TGF-β1 protein. After PN12 (left) or PN16 (right) GSCs was transfected with control or siRNA targeting IDH1 or with IDH1 R132H mutant, expression of TGF-β1 protein was analyzed by Western blotting.

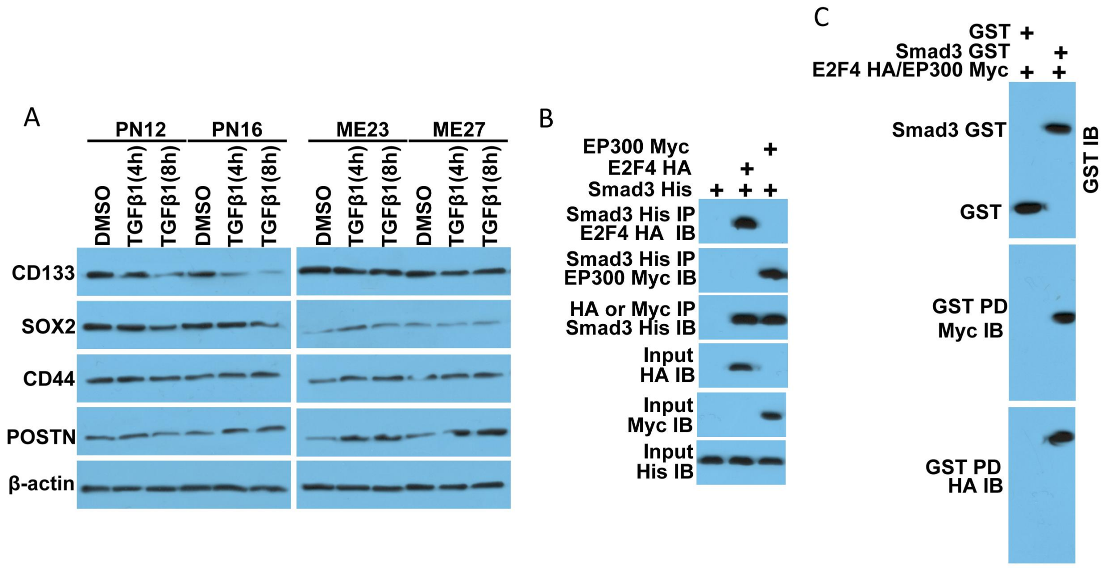

Figure S2. Competitive interaction of E2F4 and EP300 with Smad3. (**A**) Western blotting results showing the molecular subtype marker expression in proneural GSCs (PN12, PN16, and PN19), and mesenchymal GSCs (ME23, ME27, and ME29) with or without TGF-β1 treatment for 4 h or 8 h. Proneural markers: CD133 and SOX2. Mesenchymal markers: CD44 and POSTN. (**B**) Lysates from HEK293 cells expressing His-Smad3 and Myc-EP300 (or HA-E2F4) were subjected to IP followed by IB with anti-His, anti-HA and anti-Myc antibodies. (**C**) GST pull down utilizing purified GST-Smad3 and HA-E2F4 (or Myc-EP300)-expressing HEK293 cell lysates, followed by IB with anti-HA and anti-Myc antibodies. IP, immunoprecipitation; IB, immunoblotting.

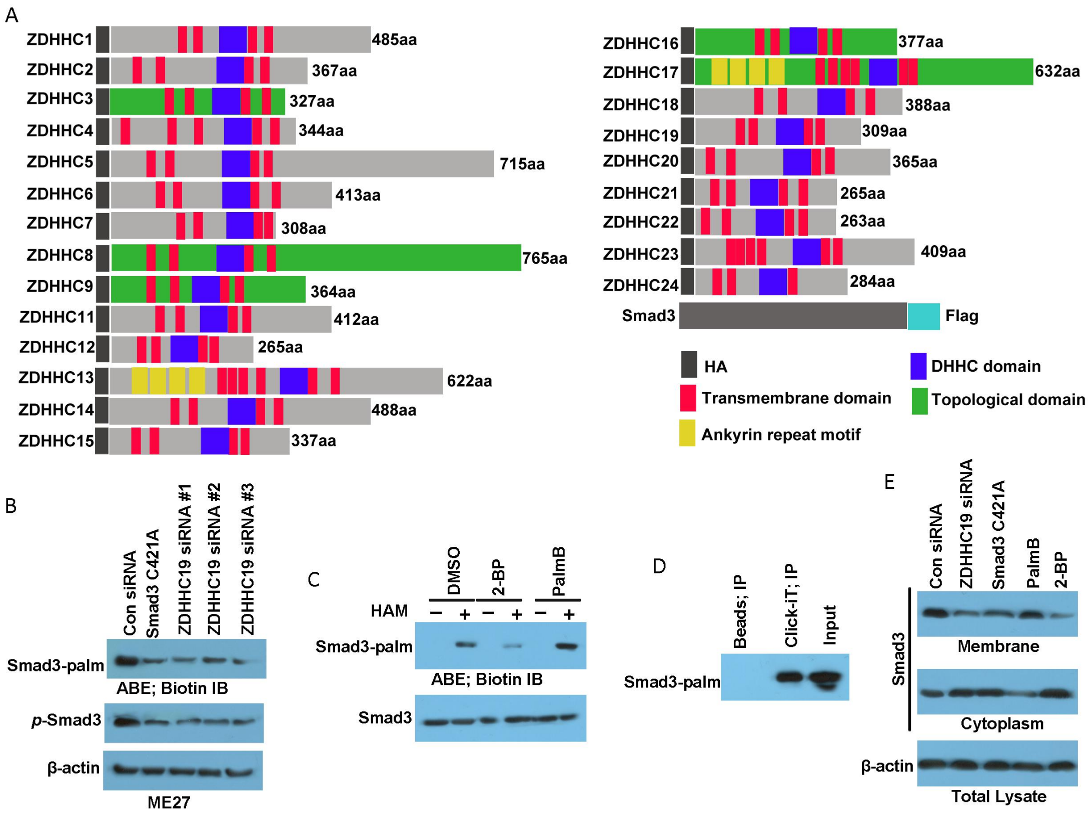

Figure S3. Smad3 palmitoylation is beneficial for activation of the TGF-β/Smad-signaling pathway. (**A**) Domain structure of human DHHC proteins and Smad3 protein. (**B**) Palmitoylation and phosphorylation of Smad3 in ME27 cells transfected with Smad3 C421A mutant constructs or infected with ZDHHC19 siRNA for 48 h. (**C**) Palmitoylation of Smad3 in ME23 cells treated with 2-BP (a palmitoylation inhibitor) or PalmB (a de-palmitoylation inhibitor) for 24 h. (**D**) ME23 cells were cultured in medium containing 100 μM palmitic acid-azide for 6 h and the cell lysates were prepared for the Click-iT reaction, followed by immunoblotting with the Smad3 antibody. (**E**) Western blot analyses of Smad3 distribution in ME23 cells transfected with ZDHHC19 siRNA, Smad3 C421A mutant plasmid, or treated with the de-palmitoylation inhibitor, PalmB (1 μM) for 48 h. HAM, hydroxylamine; IP, immunoprecipitation.

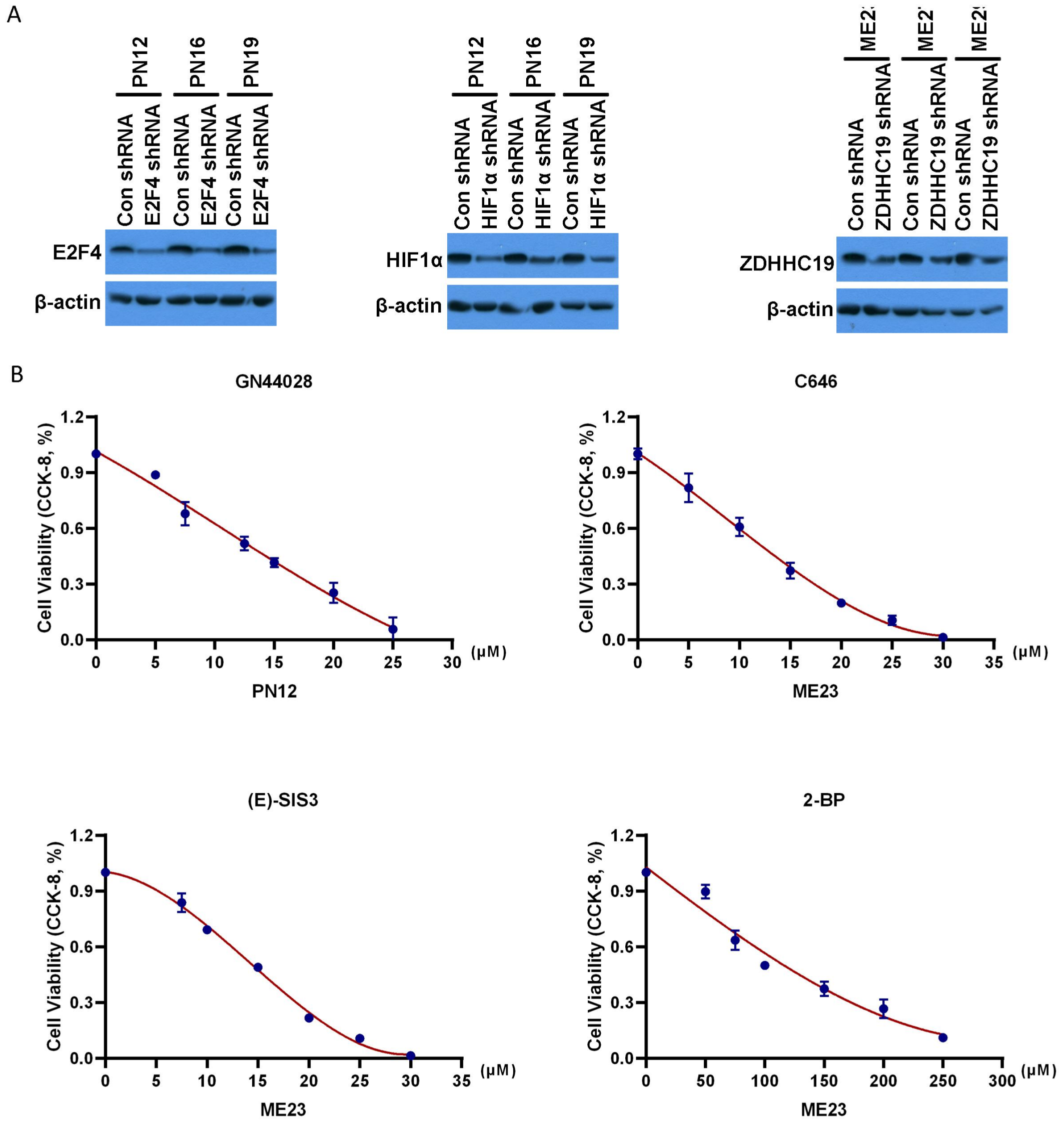

Figure S4. Inhibitory effect of GN44028, 2-BP, C646, and (E)-SIS3 in proneural GSCs and mesenchymal GSCs. (**A**) Western blot analyses of E2F4 or HIF-1α in proneural GSCs (PN12, PN16, and PN19) transfected with E2F4 shRNA or HIF-1α shRNA, and ZDHHC19 in mesenchymal GSCs (ME23, ME27, and ME29) transfected with ZDHHC19 shRNA. (**B**) Viability of proneural PN12 GSCs treated with GN44028 at the indicated concentration for 24 h. (**C**) Viability of mesenchymal ME23 GSCs treated with C646, 2-BP, or (E)-SIS3 at the indicated concentration for 24 h.

A

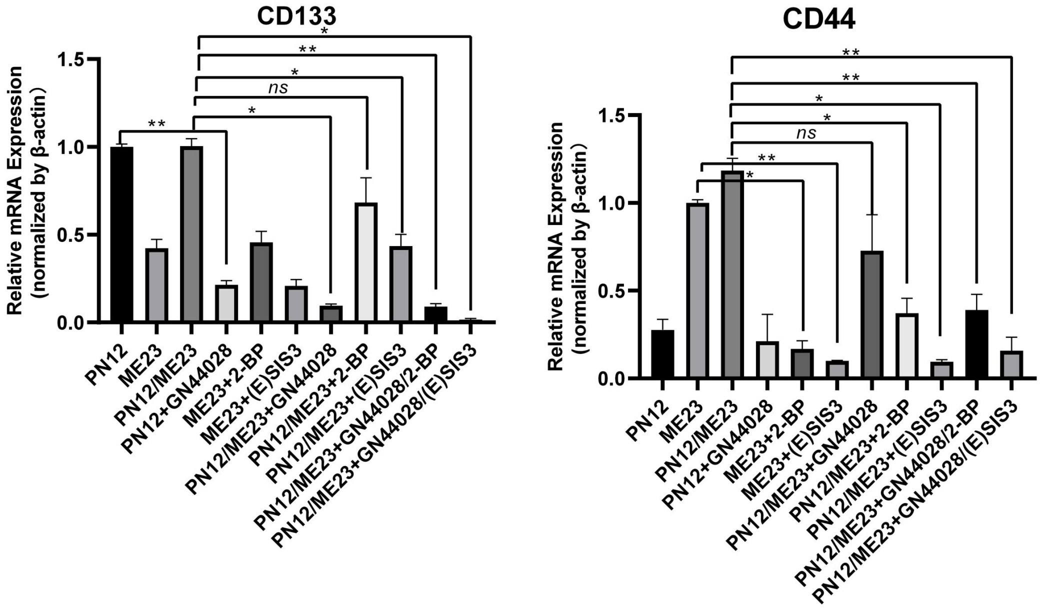

B

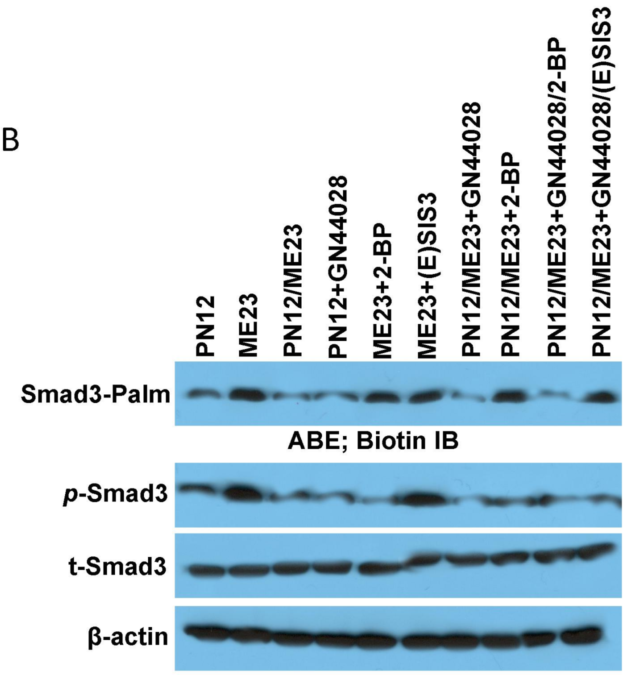

Figure S5. Change in molecular subtype *in vivo*. (**A**) Results of real time polymerase chain reaction analysis showing the molecular subtype marker expression (proneural markers, CD133; mesenchymal markers, CD44) in mice bearing a single subtype of tumor and mice bearing a mixed orthotopic tumor with PN12 and ME23 cells treated with 10 mg/kg GN44028, 7.5 mg/kg 2-BP, and 15 mg/kg (E)-SIS3 alone or in combination (*n*=5 per group) after 6 weeks. (**B**) Results of acyl-biotin exchange and Western blotting showing palmitoylation or phosphorylation of Smad3 in mice

bearing a single subtype of tumor and mice bearing a mixed orthotopic tumor with PN12 and ME23 cells treated with 10 mg/kg GN44028, 7.5 mg/kg 2-BP, and 15 mg/kg (E)-SIS3 alone or in combination (*n*=5 per group) after 6 weeks.
